# Fabrication of a conjugated microporous polymer membrane and its application for membrane catalysis

**DOI:** 10.1038/s41598-017-13827-w

**Published:** 2017-10-19

**Authors:** Jieun Lee, Jong Gil Kim, Ji Young Chang

**Affiliations:** Department of Materials Science and Engineering, College of Engineering, Seoul National University, Seoul, 08826 Korea

## Abstract

A flexible and free standing conjugated microporous polymer (CMP) membrane was prepared using a polyvinylpyrrolidone (PVP) electrospun membrane as a template. The PVP nanofibers of the template membrane were coated with a thin layer of the CMP through the *in situ* Sonogashira-Hagihara coupling reaction of 1,3,5-triethynylbenzene and 1,4-diiodobenzene. The PVP nanofibers were removed by the solvent extraction to produce the CMP membrane, which retained the entangled fibrous structure of the template membrane. Each fiber showed a hollow tubular structure having a CMP wall with a thickness of tens of nanometers. The microporous polymer membrane exhibited a high BET surface area with hierarchical porosity and good permeability. As a catalytic CMP membrane, the Ag nanoparticle-immobilized microporous polymer membrane was fabricated using an electrospun PVP@Ag membrane as a template. After being coated with the CMP, the PVP nanofibers were removed by the solvent extraction, but the Ag nanoparticles were trapped in the microporous polymer shell. The catalytic CMP membrane was successfully used for the catalytic reduction reaction of 4-nitrophenol. The hollow tubular structure and hierarchical porosity of the membrane allowed for the reactants to easily penetrate into the CMP wall and to contact the Ag nanoparticles, resulting in the high catalytic activity.

## Introduction

Porous polymer films have a broad range of applications as membranes such as in fuel cells, sensors, drug delivery systems, and catalysis in addition to their conventional uses in filtration and separation of liquids and gasses^1–6^. Porous polymer films can be classified into microporous, mesoporous, and macroporous films depending on the pore sizes. Various methods have been developed for their fabrication, including phase inversion, interfacial polymerization, and stretching^7^, but it is still challenging to prepare the films with a high volume of micropores with a diameter of smaller than 2 nm.

Microporous organic polymers are an important class of porous materials having large specific surface areas, high physical stabilities, and tunable pore structures^8–10^. However, their practical applications are considerably limited due to poor processability. Most microporous organic polymers are prepared as insoluble powders by the coupling reactions of multifunctional monomers.

Herein we report the fabrication of a conjugated microporous polymer membrane (CMP membrane) and its potential application for catalysis. The CMP membrane comprised of hollow tubular fibers was prepared using an electrospun nanofiber membrane as a sacrificial template. Electrospinning is a simple and versatile technique to fabricate a membrane consisting of continuous fibers with a diameter of hundreds of nanometers^11^. Electrospun nanofiber membranes have macropores and are used as a screen filter to separate particulates with sizes larger than several hundreds of nanometers^12,13^.

The CMP membrane had hierarchical porosity, where macropores inherited from the nonwoven electrospun nanofiber membrane and meso- and micropores of the microporous organic polymer coexisted. Each fiber showed a hollow tubular structure having a microporous organic polymer wall with a thickness of tens of nanometers. One of the promising applications of the CMP membrane is its use for membrane catalysis^14^. Microporous organic polymers have been considered as good supporting materials of heterogeneous catalysts^15–18^. In this study, a catalytic CMP membrane embedded with silver nanoparticles was prepared and its catalytic activity was evaluated in the reduction reaction of 4-nitrophenol. The reaction occurred when the reactants passed through the membrane, allowing the easy control of contact time between the reactants and the catalyst.

## Results

### Synthesis of the CMP membrane

**Figure 1:**
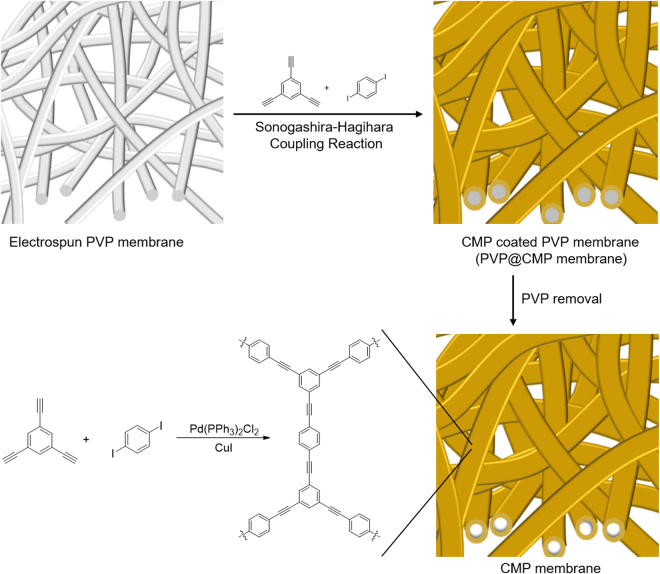
Synthetic scheme of the tubular CMP membrane.

The fabrication of the CMP membrane is summarized in Fig. 1. Firstly, an electrospun membrane was prepared as a sacrificial template by electrospinning a PVP solution (10 wt%) in DMF/ethanol (1:1, w/w); and then Sonogashira-Hagihara coupling reaction of 1,3,5-triethynylbenzene and 1,4-diiodobenzene^19^ was carried out in the presence of the PVP membrane to produce the CMP coated PVP membrane (PVP@CMP membrane). Finally, the PVP nanofibers and metal catalyst residues were removed by the solvent extraction from the PVP@CMP membrane to produce the CMP membrane^20,21^.

We chose PVP as a base polymer for electrospinning. PVP dissolved in several polar organic solvents, but was insoluble in a toluene/TEA co-solvent where Sonogashira-Hagihara coupling reaction took place. This was necessary for the electrospun PVP membrane to maintain its entangled fibrous morphology during the reaction. Moreover, the Pd catalyst could be complexed with the carbonyl groups of PVP to initiate the growth of CMP on the PVP nanofiber surface^22–24^.

### Characterization of the CMP membrane

**Figure 2:**
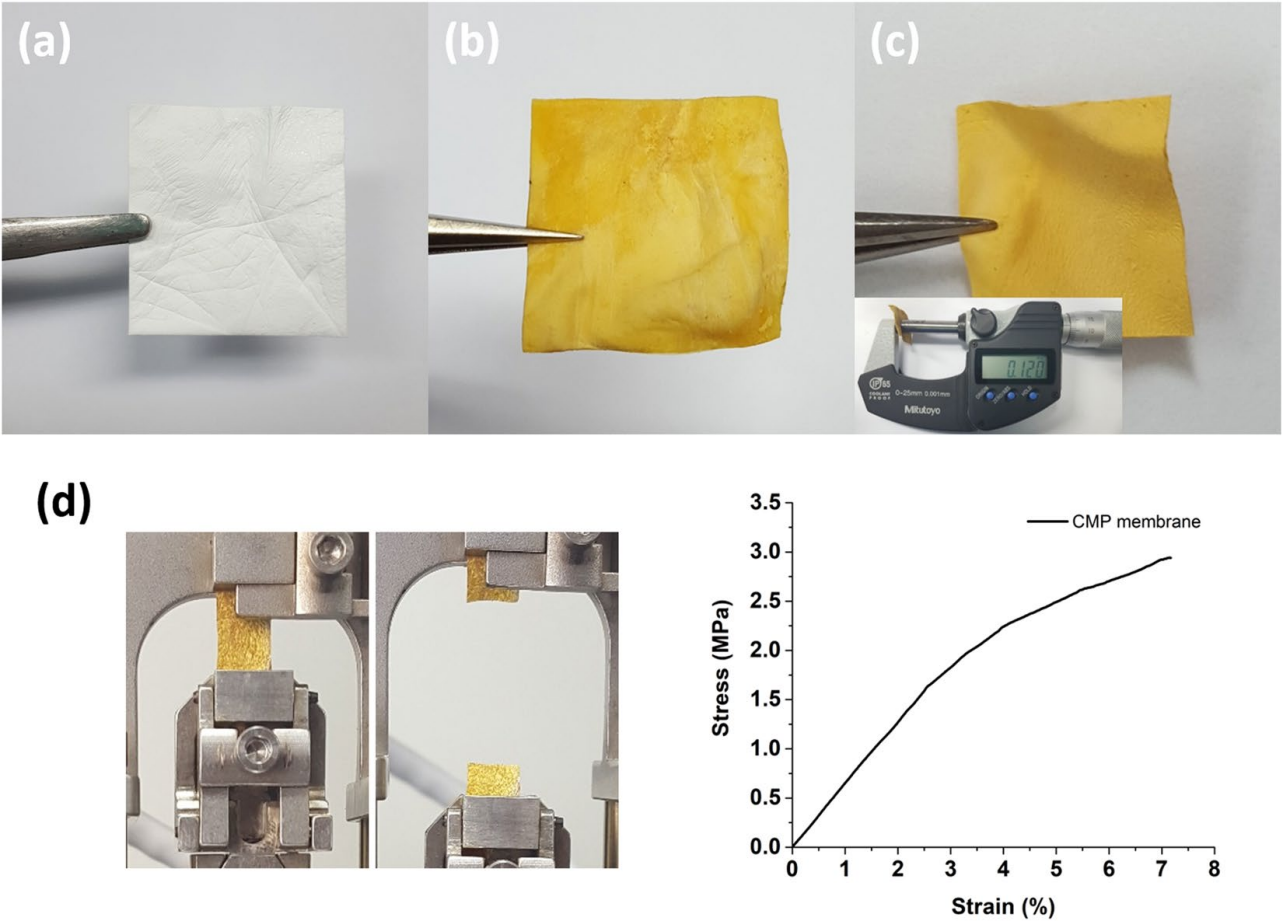
Photographs of (**a**) the electrospun PVP membrane, (**b**) the PVP@CMP membrane, and (**c**) the CMP membrane (the thickness of the CMP membrane was about 100 µm). (**d**) The CMP membrane specimen for the tensile test and the stress-strain curve.

The electrospun PVP membrane appeared white and became yellowish after being coated with the CMP (Fig. 2a,b). The PVP nanofibers and metal catalyst residues were extracted with methanol to give the CMP membrane with a thickness of about 100 µm (Fig. 2c). The tensile stress-strain curve showed that the CMP membrane could be elongated up to 7% strain and was fractured under 3 MPa tensile stress (Fig. 2d). Young’s modulus of the CMP membrane was calculated to be 63 MPa.

**Figure 3:**
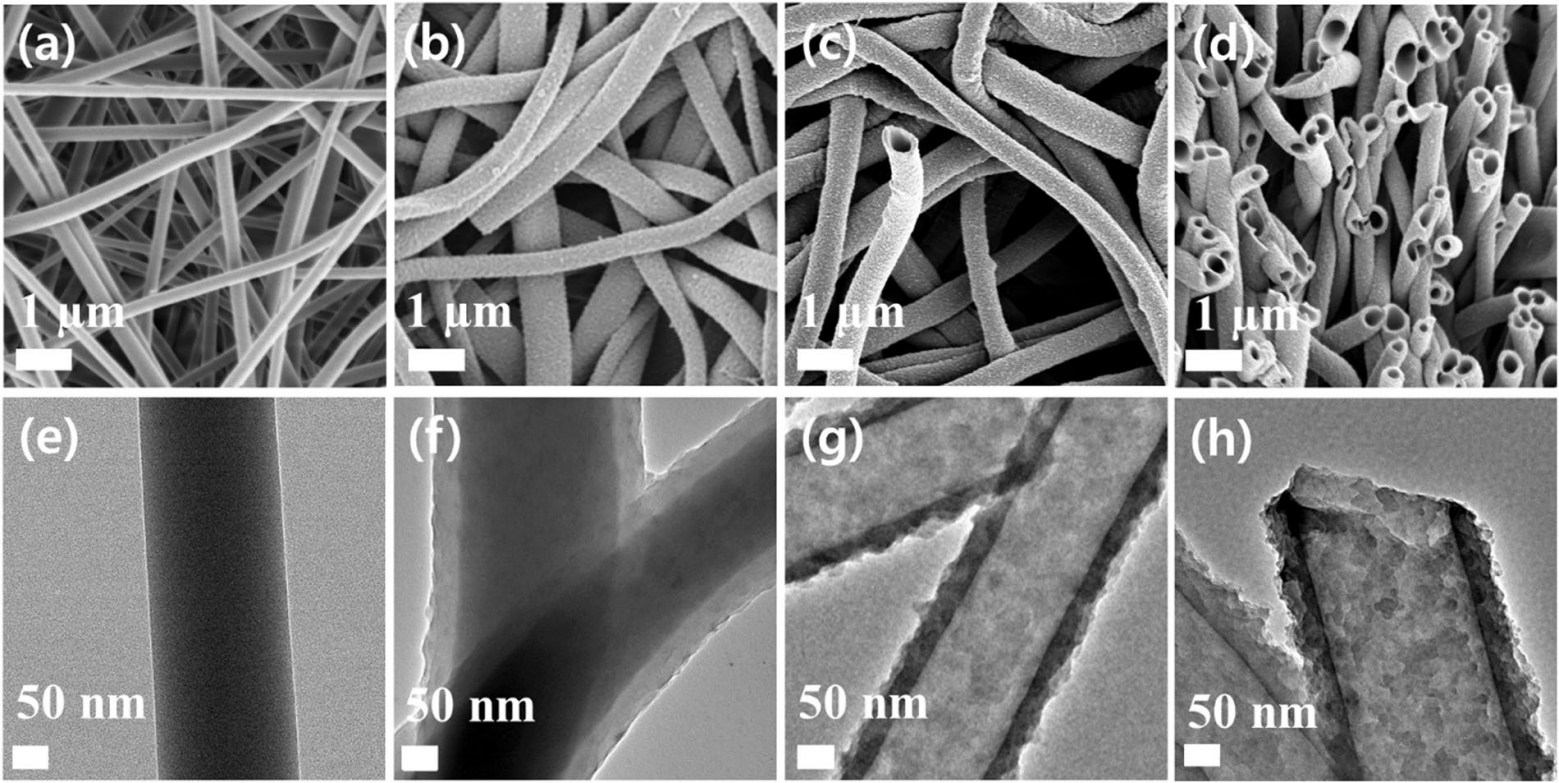
SEM images of (**a**) the electrospun PVP membrane and (**b**) the PVP@CMP membrane. SEM images of (**c**) the CMP membrane and (**d**) its cross-section. TEM images of (**e**) the electrospun PVP membrane, (**f**) the PVP@CMP membrane, and (**g**,**h**) the CMP membrane.

The fibrous microstructures of the membranes were observed by electron microscopy. The electrospun PVP nanofibers had a smooth surface with an average diameter of 200 nm (Fig. 3a,e). The CMP coated PVP membrane (PVP@CMP membrane) showed thicker nanofibers with an average diameter of 300 nm than those of the PVP membrane (Fig. 3b). The core-shell structure of the nanofibers was clearly shown in the TEM image, where the PVP nanofibers were evenly coated with the CMP shell with a thickness of about 50 nm (Fig. 3f). The SEM and TEM images in Fig. 3c,d,g,h, respectively, show a fibrous tubular structure of the CMP membrane. The average inner diameter and wall thickness of the hollow tubes were comparable to the sizes of the PVP core and the CMP shell of the PVP@CMP membrane, respectively, indicating that most PVP nanofiber cores were removed.

The growth of the CMP on the PVP nanofibers was further confirmed by solid state^13^C CP/MAS-total suppression of spinning sidebands (CP/MAS-TOSS) NMR spectroscopy (Fig. S1). The CMP powders prepared in the absence of the PVP membrane showed carbon peaks at 123 ppm and 131 ppm corresponding to the aromatic carbons bonded to acetylene carbons and hydrogen, respectively^8,20^. The acetylene carbon peak appeared at 90 ppm. In the^13^C NMR spectrum of the PVP@CMP membrane, all characteristic carbon peaks from the CMP were observed in addition to the peaks at 176, 42, 31, and 18 ppm from the core PVP^25^. The FT-IR analysis of the CMP powders showed the acetylene -C≡C- stretching vibration and aromatic C = C stretching vibration peaks at 2202 cm^−1^ and 1580 cm^−1^, respectively (Fig. S2a). In the FT-IR spectrum of the PVP@CMP membrane, the peaks from the CMP together with the peaks from PVP including the carbonyl stretching peak at 1663 cm^−1^ appeared. After the dissolution of the PVP cores, the peaks from PVP almost disappeared^26^. The CMP membrane was thermally stable, showing an initial degradation temperature of 360 °C and a high char yield over 70% at 800 °C, when determined by TGA (Fig. S2b). The char yields of PVP and the PVP@CMP membrane were 5 and 13%, respectively.

**Figure 4:**
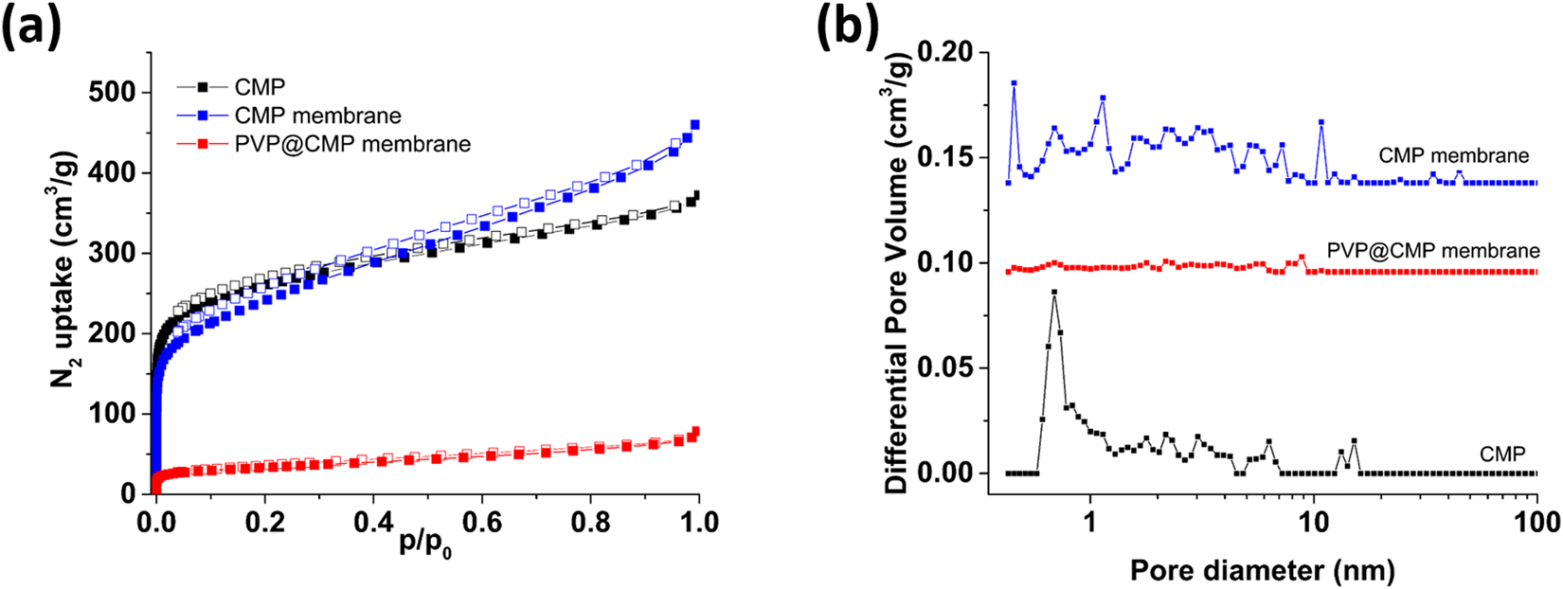
(**a**) N_2_ absorption-desorption isotherms and (**b**) NL-DFT pore size distributions of the CMP powders, the PVP@CMP membrane and the CMP membrane measured at 77 K.

The porosity of the membrane was investigated by nitrogen adsorption-desorption analysis at 77 K (Fig. 4a). The PVP@CMP membrane showed a BET surface area of 120 m^2^/g. After removing the PVP nanofibers, the surface area increased to 758 m^2^/g due to the formation of the hollow tubular structure mainly consisting of the CMP. The surface area of the CMP membrane was slightly lower than that of the CMP powders (950 m^2^/g) probably due to the presence of more macropores and a trace of remaining PVP. In the NL-DFT pore size distribution analysis, the CMP powders and the CMP membrane showed a high population of small pores with a size of less than 10 nm, while the PVP@CMP membrane had a very low pore volume because of non-porous PVP nanofibers (Fig. 4b).

**Figure 5:**
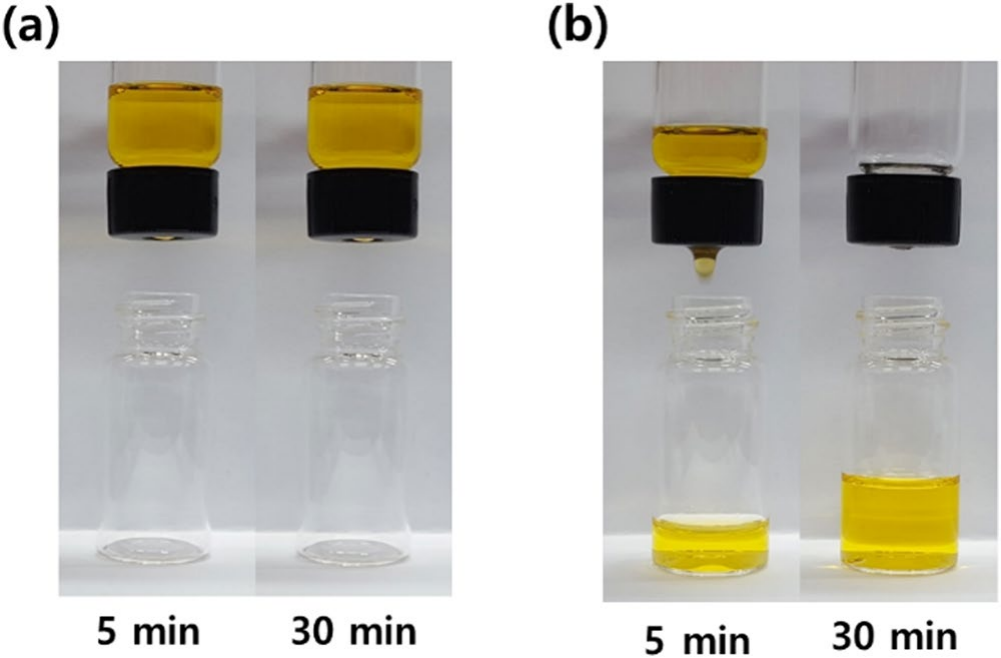
The difference in the acetone permeability of (**a**) the PVP@CMP membrane and (**b**) the CMP membrane. Acetone was dyed with sudan I (0.1 mM).

The permeability tests of the membranes were conducted using a simple experiment set-up consisting of a vial with a pinhole at the bottom and a perforated cap. An organic solvent such as acetone, THF, and hexane which did not dissolve PVP, was put into the vial and the vial mouth was tightly covered with a test membrane using the cap. The solvent was allowed to gravity flow by turning the vial upside down and the flow characteristics of the membrane was examined (Fig. S3). Figure 5 shows the difference in the permeability of the PVP@CMP membrane and the CMP membrane to acetone. The solvent could not permeate through the PVP@CMP membrane, where the PVP nanofibers seemed to impede the solvent flow, but it could pass through the CMP membrane.

### Synthesis and characterization of the CMP@Ag membrane

The high permeability and hierarchical porosity of the CMP membrane would make it a promising candidate for membrane catalysis. We prepared an Ag nanoparticle-immobilized CMP membrane and examined the feasibility of its use as a catalytic membrane. The Ag nanoparticle-immobilized CMP membrane was fabricated in the same manner as the CMP membrane except that a mixture of PVP and Ag nanoparticles in ethanol/water (4/1, w/w) was used for electrospinning (Fig. S4). The PVP containing Ag nanoparticles was prepared by dissolving PVP and silver nitrate in DMF. The Ag^ +^ ions were reduced by DMF^27^.

**Figure 6:**
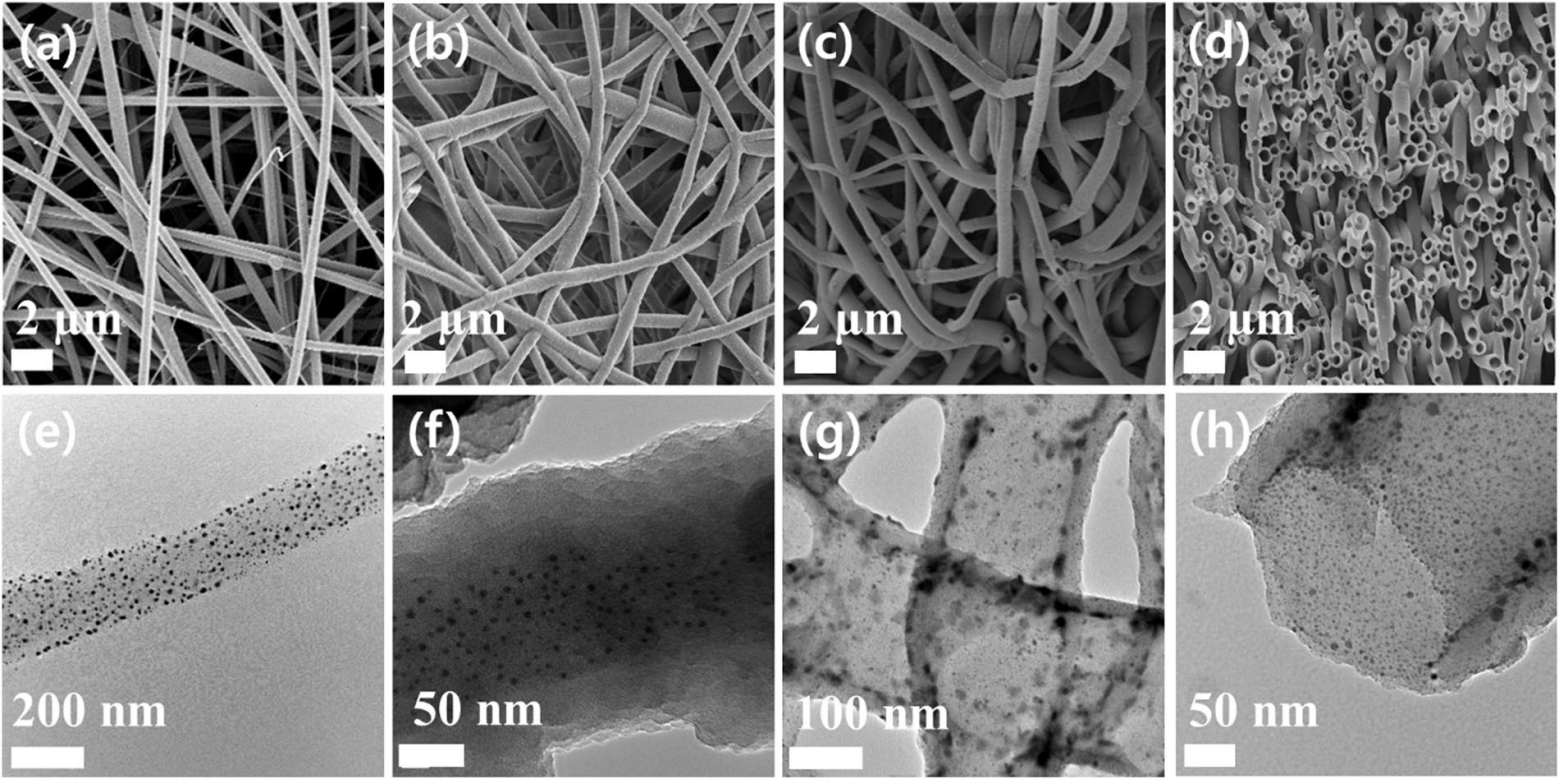
SEM images of (**a**) the electrospun PVP@Ag membrane and (**b**) the CMP coated PVP@Ag membrane. SEM images of (**c**) the CMP@Ag membrane and (**d**) its cross-section. TEM images of (**e**) the electrospun PVP@Ag membrane, (**f**) the CMP coated PVP@Ag membrane, and (**g**,**h**) the CMP@Ag membrane.

Figure 6 shows the SEM and TEM images of the electrospun PVP@Ag membrane, the CMP coated PVP@Ag membrane and the CMP@Ag membrane. The SEM image of each membrane was similar to that of the corresponding CMP membrane in Fig. 3. However, the TEM image of the electrospun PVP@Ag membrane clearly showed that Ag nanoparticles with a size of about 10 nm or less were well distributed in the PVP nanofiber (Fig. 6e). The Ag nanoparticles stayed inside the PVP nanofiber of the CMP coated PVP@Ag membrane (Fig. 6f), but they were found in the CMP wall after the PVP extraction (Fig. 6g,h). We presumed that the Ag nanoparticles were moved into the CMP shell and trapped in the pores during the extraction process of the PVP nanofibers. Some Ag nanoparticles in the CMP@Ag membrane appeared larger than those in the electrospun PVP@Ag membrane, suggesting the possible aggregation of the nanoparticles.

**Figure 7:**
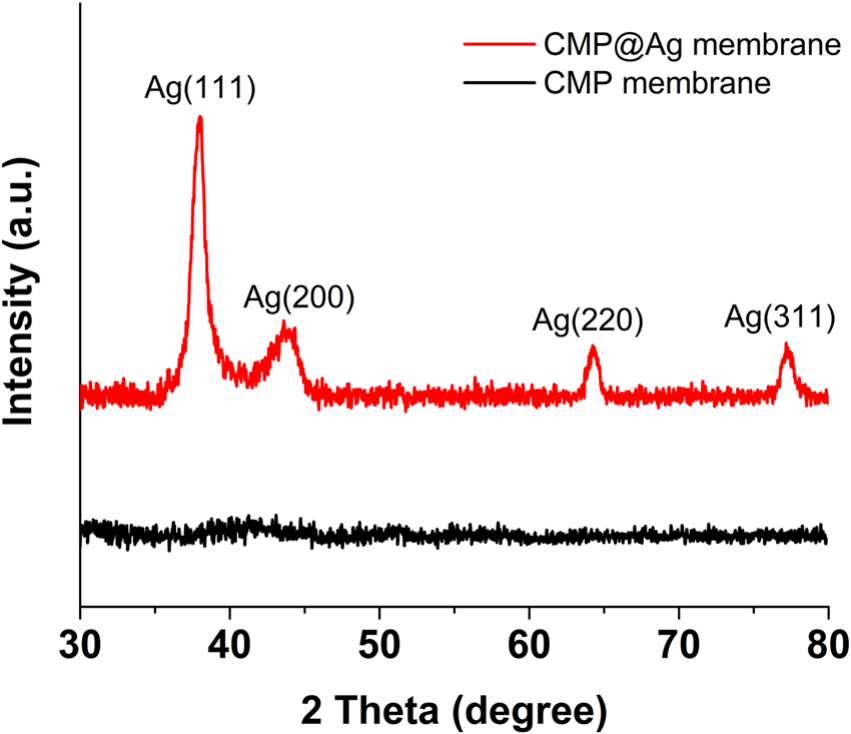
XRD patterns of the CMP membrane and the CMP@Ag membrane.

The XRD analysis of the CMP@Ag membrane showed four diffraction peaks at 2θ = 38, 44, 64, and 77°, which were corresponded to the (111), (200), (220), and (311) planes of cubic Ag, respectively (Fig. 7)^28^. The CMP@Ag membrane showed a BET surface area of 650 m^2^g^−1^ (Fig. S5).

**Figure 8:**
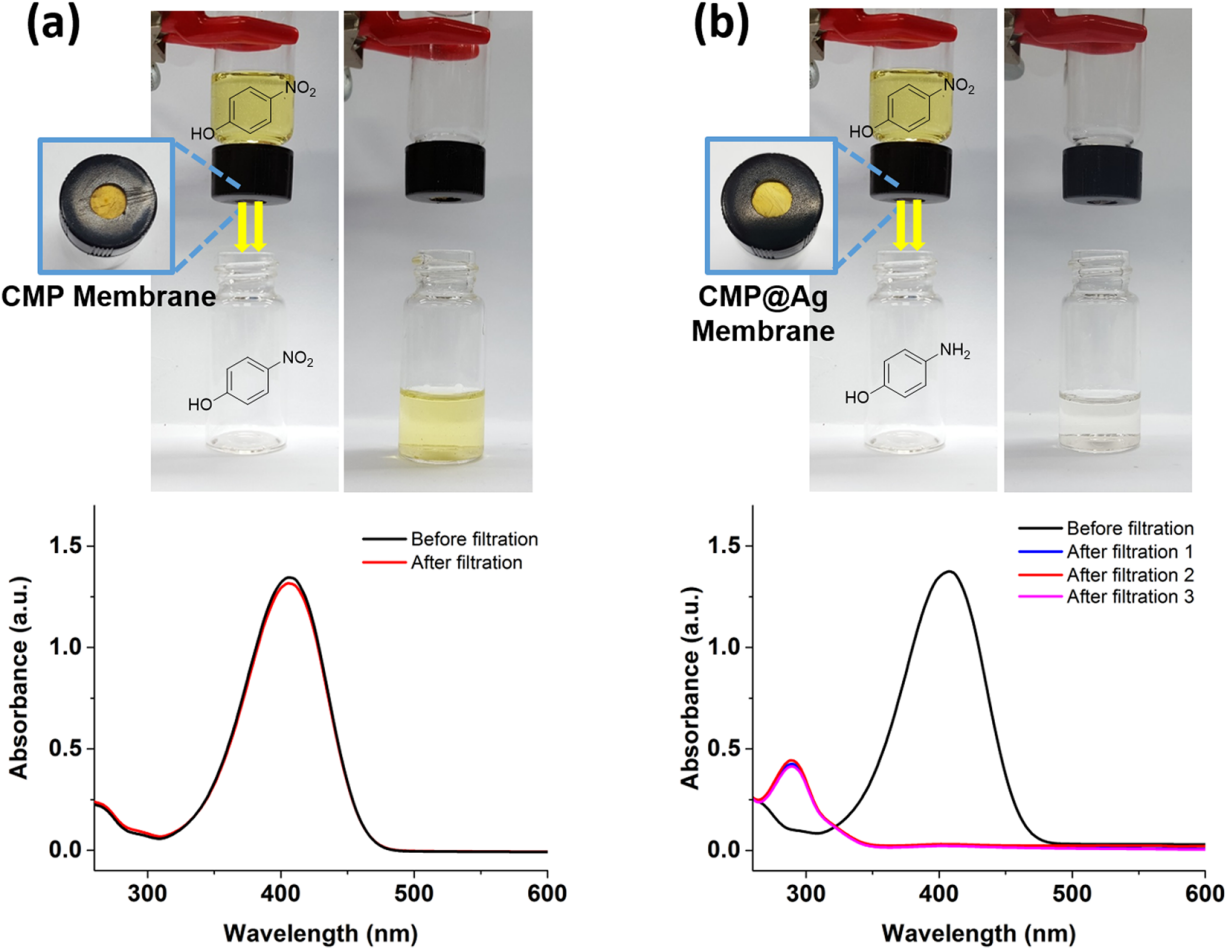
Photographs and UV-Vis absorption spectra of the reaction mixtures in the reduction reaction of 4-nitrophenol with (**a**) the CMP membrane and (**b**) the CMP@Ag membrane.

To examine the possible use of the CMP@Ag membrane for membrane catalysis, the Ag-catalyzed reduction reaction of 4-nitrophenol to 4-aminophenol was carried out in a simple membrane reactor, where the mouth of the vial was covered with the CMP@Ag membrane. A solution of 4-nitrophenol (0.1 mM) with an excess of NaBH_4_ in ethanol/water (3 mL, 50/50, w/w) was passed through the membrane by gravity flow at a rate of 0.2 mL/min (Fig. 8). The initial reaction mixture was yellowish, but became colorless after passing through the CMP@Ag membrane. A complete conversion was confirmed by UV-Vis spectroscopy as the absorption peak of 4-nitrophenol at 400 nm disappeared and a new peak from 4-aminophenol showed up at 300 nm^29^. The catalytic membrane was recycled 3 times without loss in catalytic activity. The reaction did not occur when the CMP membrane with no Ag nanoparticles was used. There was basically no change in the absorption intensity of 4-nitrophenol after the reaction mixture passed through the membrane (Fig. 8a).

## Discussion

We presented a novel method for the fabrication of the CMP membrane using the electrospun PVP membrane as a template. The template membrane was coated with the CMP by *in situ* polymerization to give the core-shell structured nanofiber membrane. The PVP cores could be easily removed from the CMP coated nanofiber membrane due to the porous structure of the CMP shell. The CMP membrane had an entangled network structure consisting of hollow tubular CMP nanofibers with a wall thickness of tens of nanometers. It had a high BET surface area, hierarchical porosity and good permeability. The catalytic CMP membrane was fabricated using Ag nanoparticle-embedded PVP nanofibers and was successfully used for membrane catalysis. The Ag nanoparticles were trapped in the CMP shell during the extraction process of the PVP@Ag nanofibers. Because of the hollow tubular structure and hierarchical porosity of the CMP membrane, the reactants could easily penetrate into the CMP wall of the CMP@Ag membrane to contact the Ag nanoparticles, resulting in the high catalytic activity. The catalysts loaded in the CMP membrane are not restricted to the Ag nanoparticle. Various metal catalytic species can be immobilized in the micro- and mesopores of the CMP membrane. Moreover, the CMP membrane can be further modified with specific functional groups by the post reaction, which will provide specific binding sites for active components of interest.

## Methods

### Materials

Polyvinylpyrrolidone (PVP, Mw = 1,300,000), silver nitrate, copper iodide, and bis(triphenylphospine)palladium(II) dichloride were purchased from Sigma-Aldrich. 1,3,5-Triethynylbenzne and 1,4-diiodobenzene were purchased from TCI. Dimethylformamide (DMF), toluene, and triethylamine (TEA) were purchased from Junsei. All chemicals were used without any further purification.

### Measurements

Solid-state ^13^C NMR spectra were recorded on a Bruker Avance II spectrometer (500 MHz) equipped with a cross-polarization/magic angle spinning (CP/MAS) probe. The FT-IR spectra were measured by a JASCO FT-IR 4200 spectrometer using KBr pellets. N_2_ adsorption-desorption isotherms were measured by a Belsorp-Max (BEL Japan, Inc.) apparatus. UV-Vis spectra were measured with a Sinco S-3150 spectrometer. Scanning electron microscopy (SEM) images were obtained by a JEOL JSM-6330F microscope. Transmission electron microscopy (TEM) images were obtained by a JEOL JEM-2010 microscope at 200 keV. Powder X-ray diffraction (PXRD) patterns were recorded on a Bruker D8 ADVANCE X-ray diffractometer (CuKα radiation, λ = 1.5418 Å). The dynamic mechanical analysis was conducted by a DMA Q800 instrument, in the tensile mode at a displacement rate of 100 µm/min. Thermogravimetric analysis (TGA) were performed using a TA modulated TGA2050 with a heating rate of 10 °C/min under nitrogen.

### Preparation of an electrospun PVP membrane

Polyvinylpyrrolidone was dissolved in DMF/ethanol (1:1, w/w) by stirring at room temperature to form a homogeneous solution with a concentration of 10 wt %. The solution was loaded in a 12 mL syringe equipped with an 18 gauge needle and electrospinning was carried out at a voltage of 20 kV and a flow rate of 1.5 mL h^−1^. A tip-to-collector distance was 20 cm^30^.

### Preparation of an electrospun PVP@Ag membrane

Silver nitrate (1.4 g) was added to a solution of PVP in DMF (50 g, 8 wt %) and was stirred for 3 h at room temperature. The Ag^+^ ions in the PVP solution were reduced to Ag nanoparticles by DMF^27^. PVP containing Ag nanoparticles was isolated by precipitation in diethyl ether, and then dried in vacuo. A 10 wt% solution of PVP containing Ag nanoparticles in ethanol/water (80/20, w/w) was loaded into a 12 mL syringe equipped with an 18 gauge needle and an electrospinning was carried out at a voltage of 22 kV and a flow rate of 1.5 mL h^−1^. A tip-to-collector distance was 20 cm.

### Preparation of the CMP membrane and the CMP@Ag membrane

A piece of electrospun PVP membrane (or PVP@Ag membrane) (100 mg), Pd(PPh_3_)_2_Cl_2_ (20 mg), and CuI (6 mg) were put into a solution of 1,3,5-triethynylbenzene (150 mg, 1 mmol) and 1,4-diiodobenzne (330 mg, 1 mmol) in a co-solvent of toluene and TEA (5:1, v/v, 30 mL). The reaction was carried out at room temperature for 24 h without stirring. The resulting composite membrane was sonicated in tetrahydrofuran for 30 min and was Soxhlet extracted with methanol for 12 h. CMP powders were prepared in the same way as described above except that an electrospun PVP membrane was not added to the reaction mixture.

### Catalytic reduction of 4-nitrophenol

A solution (3 mL) of 4-nitrophenol (0.1 mM) and NaBH_4_ (10 mM) in ethanol/water (50/50, w/w) was put into a 10 mL vial with a pinhole at the bottom and then the vial mouth was tightly covered with the CMP@Ag membrane using a perforated cap. The solution was allowed to gravity flow through the membrane at a rate of 0.2 mL/min by turning the vial upside down. The conversion of 4-nitrophenol to 4-aminophenol was determined using a UV-vis spectrophotometer.

## Electronic supplementary material

Supplementary Information
