# Supplementary material for: Fabrication of a conjugated microporous polymer membrane and its application for membrane catalysis
Source: Sci Rep. 2017 Oct 19;7:13568. doi: 10.1038/s41598-017-13827-w (PMC5648826; doi:10.1038/s41598-017-13827-w)
Supplement: Supplementary file 1 — Supplementary Information [file 41598_2017_13827_MOESM1_ESM.pdf]

Supplementary information

Fabrication of a conjugated microporous polymer membrane and its application for membrane catalysis

Jieun Lee, Jong Gil Kim and Ji Young Chang*

Department of Materials Science and Engineering

College of Engineering, Seoul National University

Seoul 08826, Korea.

E-mail: jichang@snu.ac.kr

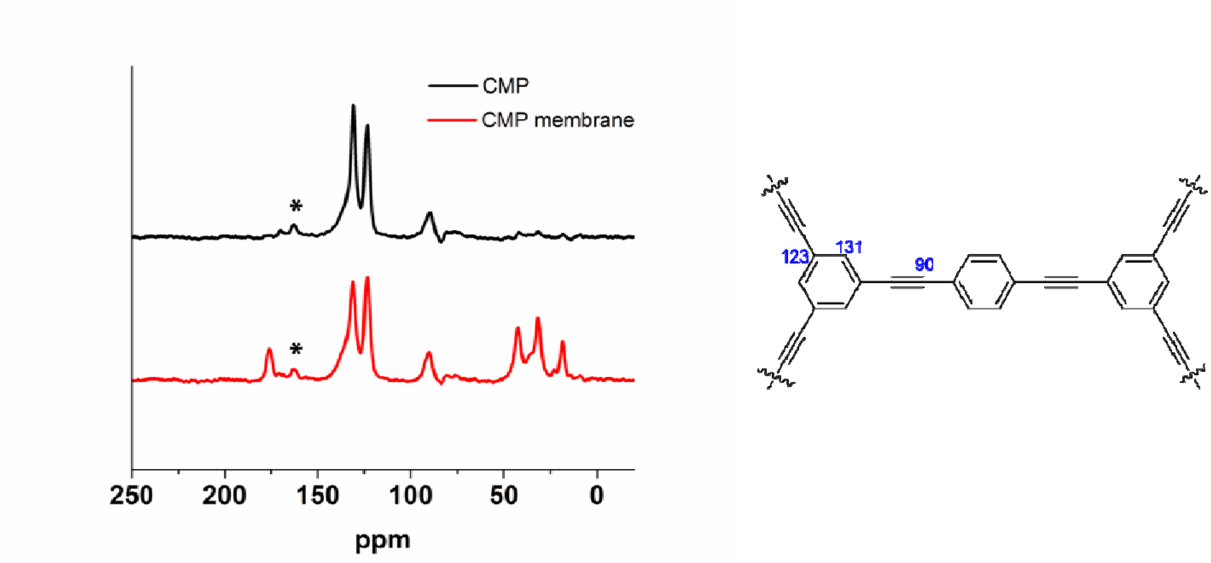

Figure S1. Solid state ^{13}C CP/MAS-TOSS NMR spectra of the CMP powders and the CMP membrane with a trace of PVP.

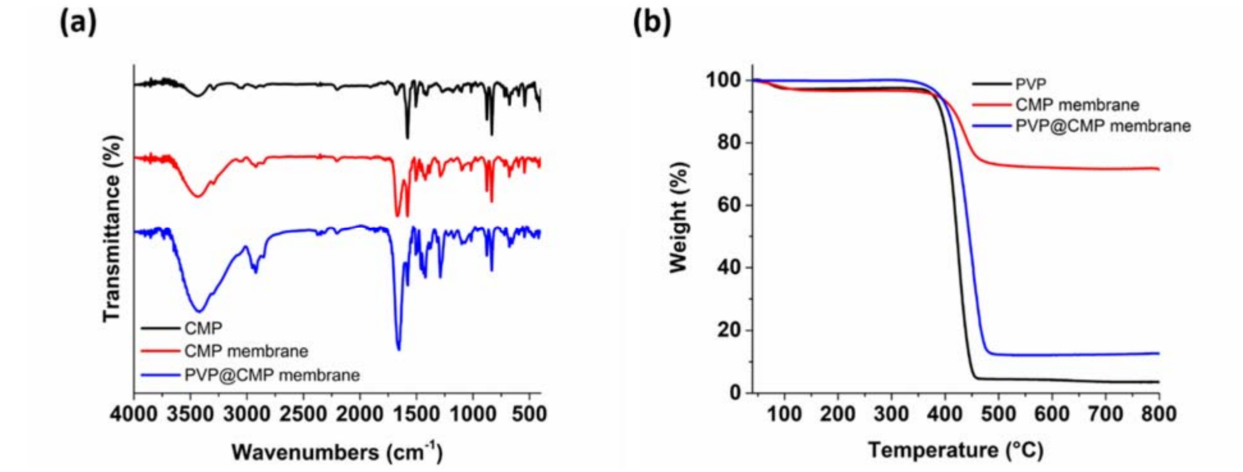

Figure S2. (a) FT-IR spectra and **(b)** TGA results of the membranes.

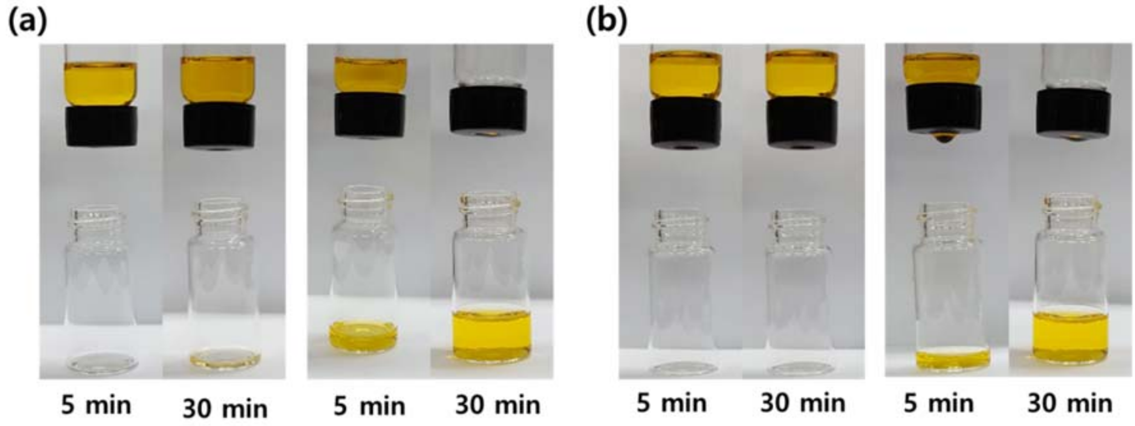

Figure S3. The permeability test results for the PVP@CMP membrane (left) and the CMP membrane (right) using **(a)** hexane and **(b)** THF.

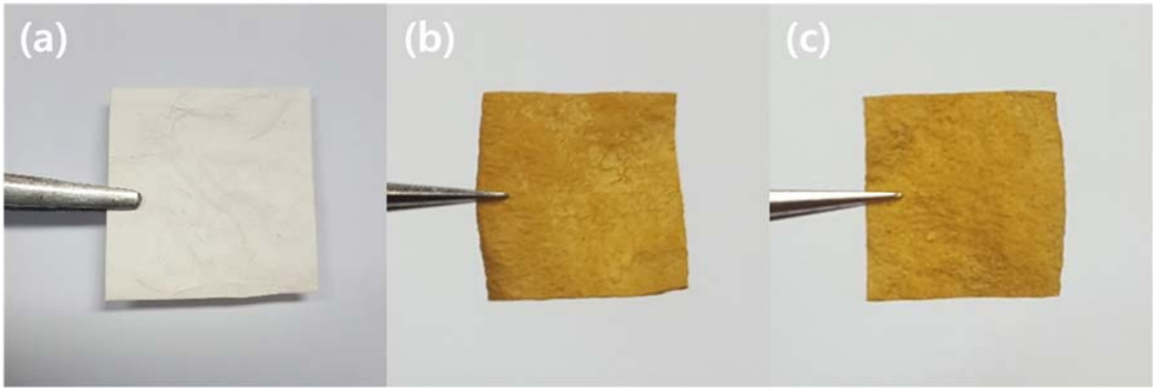

Figure S4. Photographs of **(a)** the electrospun PVP@Ag membrane, **(b)** the CMP coated PVP@Ag membrane and **(c)** the CMP@Ag membrane.

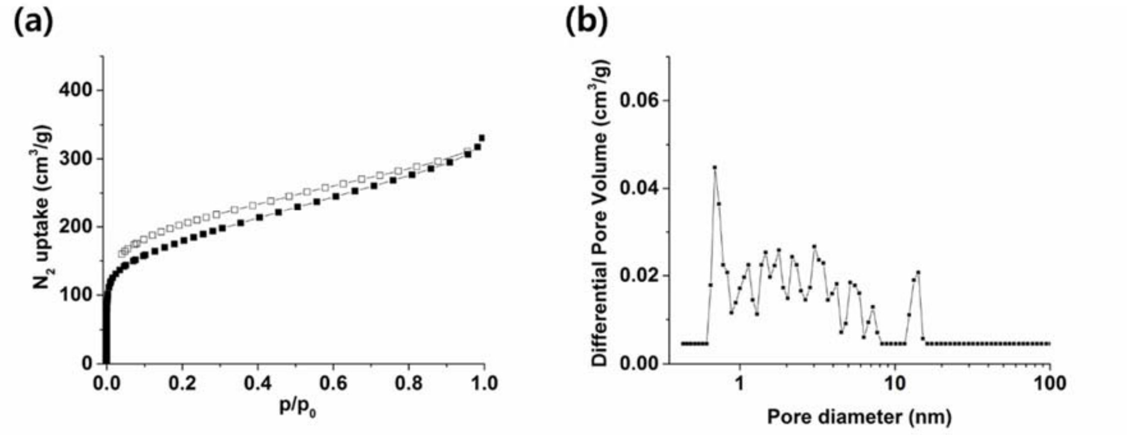

Figure S5. **(a)** N_2 absorption-desorption isotherms and **(b)** NL-DFT pore size distributions of the CMP@Ag membrane measured at 77 K.
